# The advance on pathophysiological mechanisms of type 2 chronic rhinosinusitis with nasal polyposis

**DOI:** 10.3389/falgy.2025.1599797

**Published:** 2025-07-02

**Authors:** Cheng Yang, Ling Guo, Yuhan Wang, Wenjing Jiang, Sijia Chen, Qingjia Gu

**Affiliations:** 1 Department of Otolaryngology Head and Neck Surgery, Sichuan Provincial People's Hospital, University of Electronic Science and Technology of China, Chengdu, China; 2 Department of Otolaryngology Head and Neck Surgery, Sichuan Provincial People's Hospital, Chengdu University of Traditional Chinese Medicine, Chengdu, China

**Keywords:** chronic rhinosinusitis with nasal polyposis (CRSwNP), type 2 T helper cells (Th2), type 2 innate lymphoid cells (ILC2s), epithelial barrier dysfunction, biologics

## Abstract

**Purpose:**

This review aims to explore the pathophysiological mechanisms and emerging therapies for type 2 chronic rhinosinusitis with nasal polyps (CRSwNP), driven primarily by type 2 inflammation.

**Search methods:**

A comprehensive search of relevant literature was performed in databases including PubMed, Web of Science, and Scopus, using keywords such as “chronic rhinosinusitis with nasal polyps,” “type 2 inflammation,” “Th2 cells,” “ILC2s,” “epithelial barrier dysfunction,” and “biologics”. The search was limited to articles published from January 2010 to February 2025.

**Search results:**

A total of 200 articles were initially retrieved. After screening based on relevance and quality, 163 articles were selected for this review. These included 109 basic research papers, 30 clinical studies, and 24 review articles.

**Conclusions:**

Type 2 CRSwNP pathogenesis involves Th2/ILC2-IL-4/IL-13 synergy, driving eosinophilic inflammation and tissue remodeling via a self-amplifying loop. Programmed cell death protein 1 and programmed death-ligand 1 dysregulation intensifies Th2 responses. Epithelial barrier defects (via disrupted junctions and ciliary defects) and epithelial–mesenchymal transition facilitate pathogen invasion and stromal changes. M2 macrophages amplify inflammation via CCL-24 and *Staphylococcus aureus* synergy, sustaining biofilm persistence. Targeted biologics—dupilumab (IL-4Rα inhibitor) reduces polyp burden and restores smell, while mepolizumab (anti-IL-5) and omalizumab (anti-IgE) address specific endotypes. Despite therapeutic advances, biologics require real-world validation for long-term safety and cost-effectiveness.

## 1 Background

Chronic rhinosinusitis (CRS) is a common airway inflammatory disease and bothers approximately 10% population in the world (1). The continuation of nasal obstruction, purulent secretion, loss of smell, and facial ache for more than 12 weeks were considered as typical symptoms of CRS. While CRS is not life-threatening, its high global prevalence has brought about great challenges for the quality of life of patients and caused socioeconomic burden (2). Historically, based on the presence of nasal polyp (NP), CRS can be divided into CRS with NP (CRSwNP) and CRS without NP (CRSsNP). However, it is restricted for this simple classification method to explain the complexity and diversity of CRS. In the majority of CRSwNP patients (70%–90%), eosinophil-dominated inflammatory infiltrate has been observed, which is an expression of a type 2 T helper cell (Th2)-polarized immune response (3). Previously, CRSwNP in Western countries predominantly exhibited eosinophilic inflammation, whereas Asian populations showed neutrophilic predominance. However, recent studies indicate an increasing trend of eosinophilic infiltration in Asian CRSwNP patients, potentially linked to Westernized lifestyles (4–7). The EPOS-2020 consensus classifies CRS into type 2 and non-type 2 based on immunopathologic and clinical features (8).

Type 2 inflammation plays a central role in type 2 CRSwNP, characterized by Th2 cells, type 2 cytokines (IL-4, IL-5, and IL-13), type 2 innate lymphoid cell (ILC2), eosinophilic infiltration, IgE, and comorbidities such as asthma and aspirin intolerance (9). In addition, patients with type 2 CRSwNP have more clinical symptoms, high recurrence rates, and resistance to conventional medical or surgical treatment strategies (10). Given its refractory and heterogeneous nature, this review explores the pathogenesis and treatment of type 2 CRSwNP.

## 2 Synergistic interaction between Th2 cells and ILC2s in CRSwNP

### 2.1 Th2 cells

CD4 T helper cells (Th) are pivotal in adaptive immunity. Subtypes include Th1, Th2, Th17, Tfh, and regulatory T cells (Treg), defined by their cytokine profiles (11). Th2 cells mediate adaptive responses to parasites, allergens (e.g., dust mites, molds), and helminths (12). Allergen-stimulated epithelial cells release IL-25, IL-33, and thymic stromal lymphopoietin (TSLP), activating ILC2s to amplify allergic inflammation (13).

Th2-derived cytokines (IL-4, IL-5, IL-13) regulate parasitic infections, immune regulation, and multisystem homeostasis (14). These cytokines are extensively involved in the pathogenesis of allergic inflammatory diseases, including asthma, allergic rhinitis (AR), atopic dermatitis, and chronic rhinosinusitis. IL-4 and IL-13 induce B cells to promote the production of IgE and selectively activate macrophages (15, 16). IL-13 directly acts on epithelial cells and smooth muscle cells, driving mucus secretion, airway remodeling, and airway hyperresponsiveness, while IL-5 enhances eosinophil maturation, migration, and function (17, 18). Thus, IL-4, IL-5, and IL-13 are the primary effector cytokines produced by Th2 cells during type 2 immune responses. Notably, the genes encoding these three cytokines are clustered within a single genomic segment and are regulated by the locus control region (LCR) of the Rad50 gene (19).

### 2.2 Type 2 innate lymphoid cells

In recent years, in addition to adaptive immune cells, certain innate lymphoid-like cells have been recognized to play critical roles in immunity and inflammation, particularly in early immune responses to helminth infections, allergen-induced airway inflammation, and tissue repair. ILC2s are members of the innate immune system that do not rely on antigen-specific receptors [T cell receptor(TCR)/B cell receptor (BCR)] and according to transcription factors and functions, they can be classified into three subsets: ILC1s (combat viral/intracellular pathogens; dependent on T-bet; secrete IFN-γ), ILC2s (respond to parasites/allergic reactions; dependent on GATA3/RORα; secrete IL-5, IL-13, and IL-9), and ILC3s (target extracellular bacteria/mucosal homeostasis; dependent on RORγt; secrete IL-17 and IL-22) (20, 21).

ILC2s predominantly express surface markers such as CD127 (IL-7Rα), CRTH2 (CD294), CD161, and ST2 (IL-33R) (12). They originate from common lymphoid progenitors (CLPs) in the bone marrow and further differentiate in tissues such as the thymus, gut, and lungs (12). The transcription factors GATA3, RORα, and Id2 are key regulators of ILC2 development and function (22, 23). As central effector cells in type 2 immunity, ILC2s are activated via the IL-33/ST2 and IL-25/IL-17RB signaling pathways. Upon activation, they rapidly release large quantities of type 2 cytokines to amplify type 2 inflammatory responses. Although their functions partially overlap with those of Th2 cells, ILC2s lack antigen specificity (24). Due to their rapid responsiveness and widespread presence in healthy tissues, ILC2s also play essential roles in maintaining physiological homeostasis (25).

### 2.3 Th2-ILC2 crosstalk in CRSwNP

**Figure 1:**
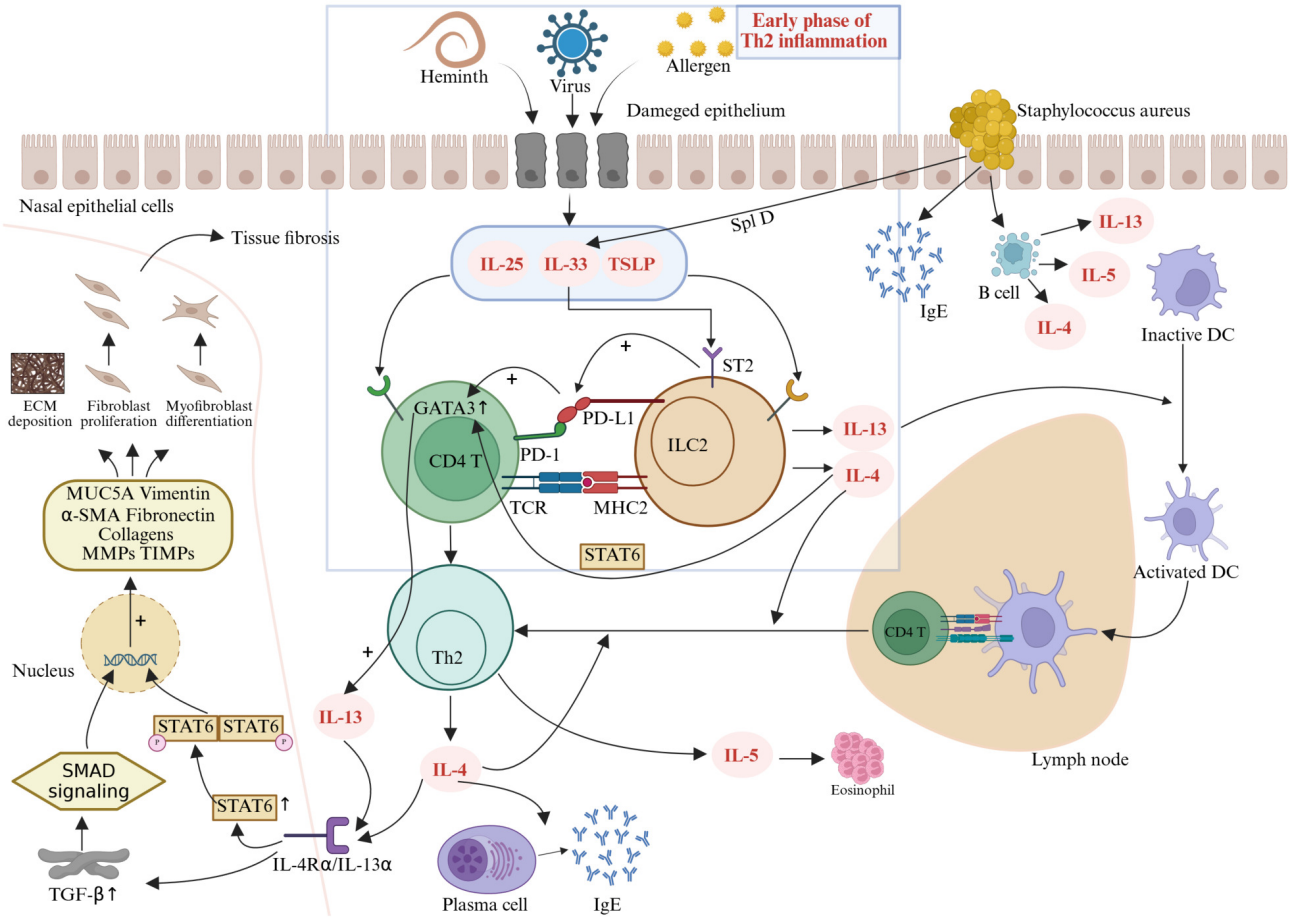
Following injury or stimulation, nasal mucosal epithelial cells release epithelial-derived alarmins such as IL-25, IL-33, and TSLP, which activate ILC2s to produce IL-4. IL-4 plays a critical role in early-phase Th2 inflammation by activating the STAT6 pathway and upregulating the transcription factor GATA3, which, in turn, amplifies the transcription of IL-4, IL-5, and IL-13 (34). IL-13 promotes the migration of activated dendritic cells from nasal polyps to draining lymph nodes, where they present antigens and prime naive CD4T cells to differentiate into Th2 cells (35). These Th2 cells secrete IL-4, IL-5, and IL-13, driving IgE production, eosinophil activation/recruitment, and tissue remodeling (29, 30). The profibrotic activities of IL-4 and IL-13 are predominantly mediated through TGF-β-dependent mechanisms (36). Binding of these cytokines to their cognate receptors triggers transmembrane JAK kinase phosphorylation cascades, promoting STAT protein homodimerization and nuclear translocation, thereby amplifying autocrine IL-4/IL-13 signaling and downstream gene expression (38, 39). Concurrently, TGF-β forms profibrotic transcriptional regulatory units via SMAD signaling complexes (40). These two pathways exhibit functional synergy in the nuclear compartment, ultimately leading to aberrant fibroblast proliferation, myofibroblast differentiation, and pathological ECM deposition, exacerbating tissue remodeling.

The pathogenesis of CRSwNP is closely linked to type 2 immune responses. ILC2s play a critical role in the formation of nasal polyps in patients with type 2 chronic rhinosinusitis. Recent studies highlight that the synergistic interaction between Th2 cells and ILC2s in driving local inflammation, tissue remodeling, and disease recalcitrance constitutes a core mechanism underlying CRSwNP progression (Figure 1). Research has demonstrated the presence of ILC2s in sinonasal mucosa, particularly in nasal polyps, where their abundance is markedly elevated (25). For instance, Stevens and Kato reported a 100-fold higher ILC2 count in nasal polyps compared with controls (26). Similarly, the study by Walford et al. revealed significantly higher proportions of ILC2s in eosinophilic nasal polyps than in non-eosinophilic polyps (27).

Type 2 CRSwNP is characterized by a Th2-polarized immune response mediated by IL-4, IL-5, IL-13, and IgE, leading to eosinophilic inflammation (28). IL-4 and IL-13 activate the IL-4Rα/STAT6 signaling pathway, inducing IgE production by B cells, promoting goblet cell hyperplasia, excessive mucus secretion, and ciliary dysfunction, thereby compromising the nasal mucosal barrier and facilitating microbial colonization (29). IL-5 binds to the IL-5Rα receptor in polyps, enhancing eosinophil maturation, recruitment, and survival, which results in pronounced eosinophilic infiltration (29). IL-13 upregulates mucin gene (MUC5AC) expression, driving mucus plug formation and fibroblast activation, which contribute to tissue fibrosis (30). Elevated IL-5 levels correlate positively with CRSwNP recurrence, and eosinophils exacerbate epithelial damage via granular proteins, perpetuating a vicious inflammatory cycle (31).

Both ILC2s and Th2 cells produce type 2 cytokines. ILC2s promote Th2 cell proliferation and differentiation through surface molecules such as MHC-II, CD80, CD86, ICOS, and OX40l (12). Conversely, Th2 cells secrete IL-4 and IL-13, which directly activate ILC2s, amplifying their proliferation and enhancing cytokine production (e.g., IL-5, IL-13) (12). This creates a positive feedback loop that intensifies inflammation. In addition, ILC2-derived IL-13 and TSLP further drive dendritic cell (DC) polarization toward a Th2 phenotype, amplifying Th2 responses (32). ILC2s also suppress Treg function, disrupting immune tolerance and exacerbating Th2 inflammation (33).

As early effectors in Th2 inflammation, ILC2s are primarily activated by epithelial-derived alarmins (IL-25, IL-33, TSLP). Activated ILC2s produce IL-4, which plays a pivotal role in the early phase of Th2 inflammation by activating the STAT6 pathway and upregulating the transcription factor GATA3 (34). GATA3 further enhances the transcription of IL-4, IL-5, and IL-13 (34). IL-13 promotes the migration of activated dendritic cells from nasal polyps to draining lymph nodes, where they present antigens and prime naive CD4 T cells to differentiate into Th2 cells (35).

Crucially, emerging evidence indicates that the profibrotic activities of IL-4 and IL-13 are predominantly mediated through the dependent mechanisms of transforming growth factor-β (TGF-β). Fichtner-Feigl et al. first revealed that IL-13 directly induces TGF-β production via specific binding to IL-13Rα2 (36). This cytokine activates SMAD signaling complexes to form profibrotic transcriptional regulatory units in the nucleus (37). Concurrently, IL-4/IL-13 binding to their cognate receptors triggers transmembrane JAK kinase phosphorylation cascades, promoting STAT6 protein homodimerization and nuclear translocation, thereby amplifying the expression of both IL-4/IL-13 autocrine signals and their downstream target genes (38, 39). Notably, these two pathways exhibit significant functional synergy in the nuclear compartment: The TGF-β/SMAD system enhances promoter accessibility of fibrogenic genes [including MUC5A, Vimentin, α-SMA, Fibronectin, Collagens, and matrix metalloproteinases (MMPs)/tissue inhibitor of matrix metalloproteinases (TIMPs)] through chromatin remodeling, while the JAK/STAT pathway maintains sustained transcriptional activation via epigenetic modifications (40). The coordinated regulatory mechanism of IL-4 and IL-13 through dual TGF-β/SMAD and JAK/STAT signaling pathways ultimately leads to aberrant fibroblast proliferation, myofibroblast differentiation, and pathological deposition of extracellular matrix (ECM), thereby exacerbating the progression of tissue remodeling.

While IL-13 orchestrates dendritic cell migration and Th2 priming, additional immune checkpoints further modulate the Th2 inflammatory cascade. Notably, the programmed cell death protein 1 and programmed death-ligand 1 (PD-1/PD-L1) axis, traditionally recognized for suppressing T-cell activation in tumor immunity (41), exhibits a paradoxical proinflammatory role in CRSwNP. This context-dependent reversal of PD-1/PD-L1 function may stem from the unique Th2-skewed microenvironment, where ILC2-Th2 cell crosstalk overrides canonical immunosuppressive signaling. Specifically, PD-L1 expressed on ILC2s interacts with PD-1 on Th2 cells, creating a feedforward loop that amplifies IL-13 release and enhances Th2 cell differentiation (42). Elevated IL-33 upregulates PD-L1 via ST2 receptors on ILC2s, and through interactions with CD4T cells, further enhances GATA3 expression, thereby driving the differentiation of these T cells into IL-13-producing Th2 cells (43). Fueled by this PD-1/PD-L1-mediated Th2 amplification, a distinct PD-1highCXCR5−CD45RA−CD4+ T-cell subset expands within nasal polyps of CRSwNP patients (44). These cells secrete elevated IL-21 and express molecules critical for T–B-cell interactions, bridging innate ILC2-driven inflammation with adaptive humoral responses. Consequently, local immunoglobulin synthesis in polyps is intensified, propelling a self-reinforcing cycle of chronic inflammation.

Within immune checkpoint regulation of Th2 cascades, the PD-1/PD-L1 axis paradoxically exerts proinflammatory effects in CRSwNP. PD-L1 expressed on ILC2s interacts with PD-1 on Th2 cells, establishing a positive feedback loop that enhances IL-13 release and Th2 cell differentiation (42). IL-33 further upregulates PD-L1 through ST2 receptors on ILC2s and amplifies GATA3 expression via crosstalk with CD4T cells, driving their differentiation into IL-13-producing Th2 subsets (43).

In addition, *Staphylococcus aureus* superantigen invasion stimulates nasal epithelial cells to produce IgE, activates B cells to upregulate IL-4/IL-5/IL-13, and increases IL-33 production through SplD, perpetuating type 2 inflammatory mechanisms (45–47).

## 3 Epithelial barrier dysfunction

The epithelial barrier serves as the first line of defense against pathogens, pollutants, and allergens (48). An intact epithelial barrier is critical for protecting the host immune system from harmful invaders (49). The nasal mucosal epithelium is primarily composed of pseudostratified ciliated columnar epithelial cells, including ciliated cells, goblet cells, and basal cells. The nasal mucosal epithelium fulfills three major functions: immune defense, mucociliary clearance (MCC), and physical barrier integrity (50). Upon allergen exposure, the nasal epithelium initiates immune responses to eliminate inflammatory cells, mitigating inflammation and disease progression (51). The coordinated beating of cilia is essential for clearing pathogens, while maintaining barrier integrity ensures normal epithelial function (52). Epithelial cells are interconnected via apical tight junctions (TJs), basolateral adherens junctions, gap junctions, and desmosomes, forming multiprotein complexes that regulate the movement of ions and macromolecules to underlying tissues and immune cells (53).

### 3.1 Immune dysfunction

When nasal epithelial cells are invaded by microbes, allergens, or irritants, they release cytokines and chemokines, leading to aberrant activation of ILC2s and upregulation of type 2 inflammatory mediators in CRSwNP. Studies indicate that Th2 cytokines IL-4 and IL-13 reduce transepithelial electrical resistance (TER) and downregulate the epithelial junctional proteins (54). Soyka et al. demonstrated that IL-4 disrupts TJs structures in human nasal epithelial cells (48). Thus, allergen-triggered cascades in CRSwNP patients result in barrier disruption, inflammatory cell infiltration, and tissue remodeling.

### 3.2 Mucociliary dysfunction

The mucociliary system is a critical defense mechanism in the nasal cavity, maintaining airway hygiene. Barrier dysfunction is often accompanied by impaired MCC, which relies on coordinated mucus secretion by goblet cells and rhythmic ciliary motion (55). Goblet cells, interspersed among ciliated cells, secrete mucus that traps pathogens, while ciliated cells, rich in apical mitochondria, generate adenosine triphosphate (ATP) to power ciliary beating (56, 57). MCC primarily functions through the coordinated beating of ciliated epithelial cells to propel mucus across the nasal mucosal surface, trapping pathogens and other inhaled irritants (58). Therefore, dysfunctional MCC will lead to bacterial colonization, mucus accumulation, and biofilm formation. Chronic infections or irritants will upregulate IL-13 and IL-17 in nasal epithelium, promoting goblet cell hyperplasia, overexpression of MUC5AC and MUC5B mucins, and ciliary dyskinesia, ultimately driving CRSwNP (59). Ostrowski et al. further showed that mice with defective cilia develop severe CRS, underscoring the role of MCC in disease progression (60). Furthermore, multiple studies have demonstrated that impaired MCC is associated with disease progression in CRS (61–63).

### 3.3 Structural barrier defects

TJs are the most paramount for epithelial integrity, blocking >90% of airborne particles and regulating immune cell permeability (64, 65). Soyka et al. observed TJs exhibit irregular immunofluorescence patterns, discontinuous expression, and reduced levels of proteins such as zonula occludens-1 (ZO-1) and occludin in CRSwNP (48). They also found decreased TER in nasal polyps (48), while Bernstein et al. reported elevated transepithelial potential and permeability in CRSwNP-derived epithelial cells (66). Li et al. confirmed severe TJs disruption in CRSwNP, with a marked downregulation of occludin, ZO-1, claudin-1, DSG1, and DSG2 (67). Huang et al. found reduced βIV-tubulin (a ciliary marker) and increased MUC5AC (a goblet cell marker), indicating epithelial remodeling via ciliary loss and goblet cell hyperplasia (68). In CRSwNP nasal polyps, epithelial cells exhibit thickened basement membranes, disrupted cilia, and reduced TJ protein expression, facilitating pathogen/allergen penetration and chronic inflammation (69). Altered mucus composition (MUC5AC overexpression) and ciliary dyskinesia further impair mucus clearance, perpetuating a vicious cycle of mucus retention (69). Collectively, epithelial barrier dysfunction plays a pivotal role in initiating and sustaining CRSwNP inflammation. Thus, therapies targeting epithelial repair, alongside conventional anti-inflammatory approaches, represent a promising future direction for CRSwNP management.

## 4 Tissue remodeling: epithelial–mesenchymal transition

Tissue remodeling in CRSwNP is a consequence of prolonged inflammatory stimulation, leading to structural alterations closely associated with persistent injury and aberrant repair. Epithelial–mesenchymal transition (EMT), a key mechanism in tissue remodeling, plays a pivotal role in postinjury chronic inflammation, wound healing, and tissue restructuring (70, 71). EMT is characterized by the loss of epithelial traits (cell polarity and intercellular junctions) and the acquisition of mesenchymal features (migratory and invasive capacities), participating in both physiological processes (e.g., embryonic development and tissue repair) and pathological conditions (e.g., cancer metastasis) (72). EMT is categorized into three types: type I EMT: involved in embryonic development; type II EMT: associated with wound healing and epithelial repair; and type III EMT: linked to cancer progression and metastasis (73). Also, type II EMT is implicated in the chronic inflammatory repair process of CRSwNP (73). During EMT, epithelial markers such as E-cadherin and ZO-1 are downregulated, while mesenchymal markers like N-cadherin and vimentin are upregulated, ultimately weakening intercellular adhesion and enhancing cell motility (74). Comparative proteomic analyses by Kao et al. of nasal mucosa from healthy individuals and CRS patients revealed heightened EMT activity in CRS tissues (75). Li et al. demonstrated reduced E-cadherin expression and elevated levels of TGF-β1, α-SMA, fibronectin, and vimentin in CRSwNP nasal epithelial cells compared with controls (76). Wang et al. further identified differential vimentin expression across CRS subgroups [controls, CRSsNP, non-eosinophilic CRSwNP (ECRSwNP), and ECRSwNP], with the highest vimentin positivity in ECRSwNP epithelium, suggesting EMT is particularly prominent in eosinophilic CRSwNP (77).

Moreover, multiple studies have documented EMT-driven tissue remodeling in CRSwNP, including polyp formation, severe basement membrane edema, albumin deposition, pseudocyst formation, and subepithelial/perivascular inflammatory infiltration (77–79). In conclusion, substantial evidence supports the critical role of EMT in CRSwNP tissue remodeling. Monitoring EMT biomarkers, such as E-cadherin and vimentin, may aid in predicting disease prognosis and recurrence.

## 5 Immune microenvironment: M2 macrophages

Macrophages, a critical component of the innate immune system, exhibit high plasticity and heterogeneity, playing pivotal roles in tumor immunity and inflammatory responses (80). Functionally similar to dendritic cells, macrophages engage in phagocytosis, antigen presentation, and cytokine production (81). They are broadly categorized into two phenotypes: M1 macrophages (classically activated), which drive proinflammatory responses, produce inflammatory cytokines, and regulate Th1/antipathogen immunity; and M2 macrophages (alternatively activated), polarized by Th2 cytokines (e.g., IL-4, IL-13, IL-10, TGF-β, M-CSF), which promote tissue repair, Th2 immunity, and anti-inflammatory responses characterized by high IL-10 and low IL-12 expression (82).

Growing evidence implicates both M1 and M2 macrophages in the pathogenesis of CRSwNP, with M2 macrophage infiltration being particularly critical in type 2 CRSwNP (83–85). M2 macrophages can be activated by IL-4, IL-13, IL-10, M-CSF, or TGF-β, triggering anti-inflammatory responses, tissue remodeling, and Th2 immune regulation (86). Zhong et al. observed elevated M2 macrophage levels in ECRSwNP nasal polyps, correlating positively with local IL-5 levels (83). Krysko et al. similarly reported increased M2 macrophages in CRSwNP, with Th2 markers [IL-5, eosinophil cationic protein (ECP), IgE] positively associated with macrophage abundance (87). Deng et al. demonstrated that M2-derived CCL-24 exacerbates eosinophilic inflammation in nasal polyps (88), while Bao et al. showed that INPP4A deficiency promotes M2 polarization, enhancing chemokine secretion and recruiting Th2 cells/eosinophils (89).In addition, M2 macrophages can regulate angiogenesis and extracellular matrix deposition, driving tissue remodeling (90). Notably, M2 macrophages amplify eosinophilic inflammation via eosinophil recruitment and release of remodeling mediators, highlighting their central role in type 2 CRSwNP (91).

### 5.1. IL-10 and M2 macrophage dynamics

IL-10, a canonical Th2 cytokine, modulates M2 polarization and dampens CRSwNP inflammation by reducing antigen presentation, proinflammatory cytokine production, and macrophage bactericidal activity (92, 93). High IL-10 expression is a hallmark of M2 macrophages, which are significantly increased in nasal polyps. However, in ECRSwNP, the number of IL-10-producing M2 macrophages is reduced, and decreased IL-10 levels may contribute to the persistence of nasal mucosal inflammation (94). Consequently, M2 macrophages exhibit diminished immunosuppressive capacity due to deficient IL-10 production in ECRSwNP. While this reduction in IL-10 activity may enhance immune activation and pathogen clearance during acute severe infections, benefiting the host, it simultaneously exacerbates the development of eosinophilic inflammation (95). Macrophage migration inhibitory factor (MIF), a pleiotropic molecule highly expressed in macrophages, enhances M2 polarization and CCL-24 secretion, promoting eosinophil accumulation and worsening disease prognosis (96, 97). Elevated serum and tissue MIF levels in CRSwNP correlate with eosinophilic inflammation severity and recurrence (98).

### 5.2. Autophagy and M2 dysregulation

Defective macrophage autophagy may drive ECRSwNP progression (99). Normal autophagy clears pathogens and damaged organelles, maintaining homeostasis. Choi et al. found reduced myeloid autophagy in ECRS mice, leading to eosinophilia, epithelial hyperplasia, and mucosal thickening (100). Autophagy-deficient macrophages increase IFN-γ and IL-1β, upregulating Th2 cytokines (IL-4, IL-5, IL-13) (101). Enhancing autophagy thus represents a potential therapeutic target. Finally, M2 macrophages can upregulate vascular endothelial growth factor, promoting epithelial proliferation, microvascular permeability, and polyp edema (102, 103). They also secrete MMPs, facilitating pseudocyst formation and polyp growth (104).

**Figure 2:**
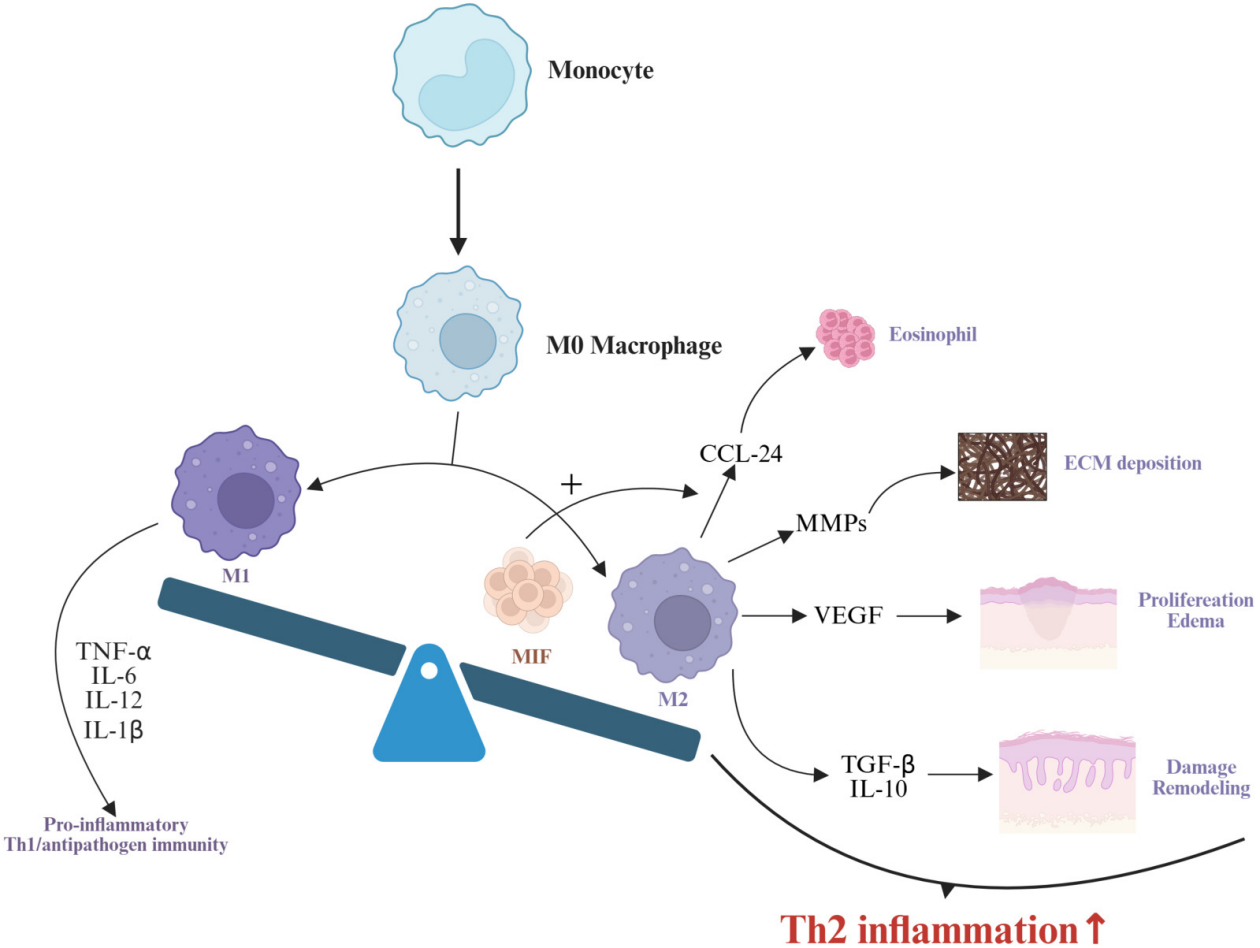
Monocytes differentiate into M0 macrophages, which further polarize into M1 and M2 macrophages. M1 macrophages drive proinflammatory responses and Th1/antipathogen immunity by secreting TNF-α, IL-6, and IL-12 (82). MIF enhances M2 macrophage polarization and promotes CCL-24 secretion by M2 macrophages (96, 97). M2 macrophages contribute to type 2 inflammation through multiple mechanisms: CCL-24 mediates eosinophil recruitment (98), MMPs participate in extracellular matrix deposition (104), VEGF promotes hyperplasia of nasal mucosal epithelial cells and interstitial edema (103), while TGF-β and IL-10 drive nasal mucosal epithelial damage and remodeling (86). Collectively, these processes synergistically amplify type 2 inflammatory responses.

In summary, M2 macrophages are central players in type 2 CRSwNP, orchestrating eosinophilic inflammation, tissue remodeling, and disease recurrence through cytokine crosstalk, chemokine recruitment, and impaired resolution mechanisms (Figure 2).

## 6 Microbial infection: *S. aureus*

*Staphylococcus aureus* produces virulence factors and enterotoxins to promote inflammation, such as *S. aureus* superantigens. This protein toxin binds directly to T-cell receptors outside the natural antigen binding site (105). This direct binding leads to an overactivation of the T-cell immune response while promoting the proliferation of B cells, leading to local production of polyclonal IgE, and hence eosinophilic activation (105). In recent years, there has been increasing evidence that *S. aureus* colonization is the initiator of disease that promotes immune disorders, the destruction of the epithelial barrier and microenvironment of bacteria, leading to the formation of biofilms and intractable diseases. Previous studies have shown that *S. aureus* has been detected in the nasal cavity in 2/3 CRSwNP patients, but only 1/3 and 1/5 *S. aureus* were colonized in CRSsNP patients and healthy controls (106, 107). In addition, *S. aureus* may play a leading role in the pathogenesis of CRSwNP in the Chinese population (108). In patients with CRSwNP, not only does *S. aureus* have an increased colonization rate, but it is also associated with mucosal infiltration and percentage of eosinophils in peripheral blood (109). In half of patients with CRSwNP, IgE antibodies containing enterotoxins from *S. aureus* can be detected in homogeneous pulp from nasal polyps (110). These patients were characterized by high eosinophilic cell infiltration, increased expression of Th2 cytokines and increased blood IgE concentration (110). Staphylococcal superantigens activate B cells to upregulate IL-4, IL-5, and IL-13, which leads to the production of IgE and the IgE antibodies of superantigens in patients with CRSwNP (45). It causes the persistence of Th2 inflammation. *S. aureus* enterotoxins damage and remodel tissues by inhibiting regulatory T cells, increasing the production of Th2 cytokines, and enhancing the function of eosinophils and mast cells (46). In addition, *S. aureus* enterotoxins stimulate endoplasmic reticulum stress and reactive oxygen production in patients with CRSwNP (111). The two combined to destroy the epithelial barrier and amplify the Th2 inflammatory response (111). This, in turn, drives the progress of CRSwNP. Serine protease-like protein (Spl) D is one of the six subtypes produced by *S. aureus*, which increases IL-33 production and promotes the Th2 immune response (47). In summary, *S. aureus* plays a vital role in the persistence of inflammation and polyp formation in type 2 CRSwNP.

## 7 The current treatment landscape and biologics for CRSwNP

At present, biologics targeting type 2 inflammation represent a novel therapeutic direction for severe and uncontrolled CRSwNP. The treatment goals for CRSwNP are categorized into symptom control, remission, and cure, but current therapies rarely achieve complete cure (112). With the emergence of biologics, this goal may now be attainable. In Europe and the United States, three monoclonal antibodies have been approved by the US Food and Drug Administration (FDA) and European Medicines Agency (EMA) for the treatment of severe, refractory CRSwNP (113). Unlike conventional medications or surgery, these biologics (humanized monoclonal antibodies) act primarily by inhibiting the type 2 cytokine cascade (114). In the past, in 2016, Stevens et al. has mentioned that omalizumab, mepolizumab, and dupilumab can reduce acute asthma attacks and that these biologics may have potentially more beneficial effects in patients with both asthma and CRSwNP (115). However, at that time, omalizumab, Mepolizumab and dupilumab were not approved for the treatment of CRSwNP. Anyway, since different biologics target distinct molecular pathways, accurate identification of disease endotypes is critical to guide precise, personalized treatment strategies.

### 7.1 Dupilumab: targeting interleukin 4 and interleukin 13

Dupilumab, an inhibitor of IL-4Rα, suppresses eosinophil migration into tissues, downregulates IgE infiltration, and concurrently blocks IL-4 and IL-13 signaling pathways (116). A phase 3 clinical trial demonstrated that after 24 weeks of treatment, Dupilumab significantly reduced nasal polyp score (NPS, ≥2), nasal congestion score (NCS, ≥1), Lund–Mackay score (LMS, ≥7), and Sino-Nasal Outcome Test-22 (SNOT-22) score (≥8.9) in patients with severe CRSwNP (117). A network meta-analysis incorporating seven randomized controlled trials (RCTs) compared the efficacy and safety of biologics for CRSwNP (118). The results showed that Dupilumab outperformed benralizumab, mepolizumab, and omalizumab at 24 weeks and follow-up endpoints, demonstrating superior reductions in NPS and nasal congestion severity (118). Hopkins et al. conducted a *post hoc* analysis revealing that dupilumab improved CRSwNP outcomes at week 24 regardless of prior surgical history, indicating its efficacy is unaffected by previous surgeries (119). Olfactory dysfunction is a refractory feature of CRSwNP, with approximately 80% of severe cases presenting anosmia, 20% hyposmia, and fewer than 5% retaining normosmia. Studies indicate that dupilumab rapidly and significantly restores olfactory function, even reversing severe type 2 inflammation-induced anosmia (120). Matsuyama et al. found that after 24 weeks of dupilumab treatment, the circulating Th1/Th2 ratio increased compared with baseline, while PD-1-positive Tregs decreased (121). Dupilumab markedly alleviates type 2 inflammation in CRSwNP. Compared with placebo, 16 weeks of treatment significantly reduced IgE and eotaxin-3 levels in nasal secretions, as well as ECP, eotaxin-2, eotaxin-3, chemokine, IgE, and IL-13 levels in nasal polyp tissues (122). With regard to safety, dupilumab exhibited no serious adverse events (AEs) over 3 years of use in patients with atopic dermatitis (123). Overall, dupilumab represents an effective therapeutic option for patients with type 2 CRSwNP.

### 7.2 Mepolizumab: targeting interleukin 5

Mepolizumab exerts its therapeutic effects primarily by inhibiting IL-5, thereby reducing eosinophil chemotaxis, differentiation, activation, and survival (124). Previous studies have demonstrated that mepolizumab effectively treats severe eosinophilic asthma (SEA), hypereosinophilic syndrome (HES), and eosinophilic granulomatosis with polyangiitis (EGPA) by lowering circulating eosinophil levels, and it also significantly reduces dependence on systemic corticosteroids (SCC) and improves prognosis in these patients (125–127). Results from a phase 3 clinical trial indicate that mepolizumab effectively decreases circulating eosinophils, nasal polyp size, nasal congestion, systemic corticosteroid use, and the need for surgery in patients with type 2 CRSwNP (128). Following 8 weeks of mepolizumab treatment, CRSwNP patients exhibited significant reductions in blood eosinophils, serum ECP, IL-5α levels, as well as nasal secretion myeloperoxidase (MPO), periostin, IL-6, IL-1β, and IL-5Rα levels (124). As mentioned earlier, VEGF promotes edema and growth in NPs, exacerbating CRSwNP progression (103). A small-scale study (*n* = 12) found that mepolizumab markedly reduces VEGF, VEGFR1, and VEGFR2 expression in middle turbinate mucosa (129). Real-life studies demonstrate significant improvements in quality of life after 6 and 12 months of mepolizumab therapy, as assessed by the SNOT-22 and Rhinosinusitis Outcomes Measure-31 (RSOM-31) (130). Mepolizumab also shows efficacy in restoring olfactory function. In a cohort of severe CRSwNP patients, 22% achieved partial improvement and 14% full recovery in olfaction (131). Regarding safety, mepolizumab exhibits a favorable profile. In one real-life study, none of the 27 patients experienced AEs, while other studies reported only two cases of serious AEs (132). Thus, mepolizumab is considered a cornerstone therapy for severe type 2 CRSwNP with elevated circulating eosinophils.

### 7.3 Omalizumab: targeting immunoglobulin E

Omalizumab binds to free IgE, disrupting its high-affinity interaction with FcɛRI on mast cells and basophils (133). This reduces IgE receptor expression on mast cells, basophils, and dendritic cells, thereby suppressing mediator release (133). In a randomized controlled trial involving 24 CRSwNP patients with comorbid asthma, omalizumab significantly improved NPS, LMS, and patient-reported outcomes, irrespective of allergy status (134). In addition, eosinophilic CRSwNP patients with asthma exhibited reduced peripheral blood eosinophils, fractional exhaled nitric oxide (FeNO) levels, and restored corticosteroid sensitivity following omalizumab treatment (135). In addition, a global, replicate phase III trial led by Bachert demonstrated that omalizumab add-on therapy to intranasal mometasone achieved statistically significant improvements in NPS, mean daily NCS, and patient-reported symptom severity assessments versus placebo at week 24 (all *P* < 0.001) (131). A real-life study reported olfactory improvement in 36% of severe asthma and CRSwNP patients treated with omalizumab (136). However, recent real-life analyses suggest a potential association between omalizumab and increased malignancy risk (137). In severe asthma populations, factors such as age, obesity, smoking, nasal polyps, and allergic rhinitis may diminish omalizumab's therapeutic efficacy (138). Anyway, evidence supporting omalizumab's efficacy in CRSwNP has been established through successful phase 3 trials (139). Omalizumab is also used for AR and other allergic conditions (140).

### 7.4 Other type 2-targeting agents

In addition to the approved dupilumab, mepolizumab, and omalizumab, there are still several biologics for type 2 inflammatory pathways that show potential in the treatment of CRSwNP but have not yet been approved by the FDA.

Among them, tezepelumab was the first monoclonal antibody to target TSLP, which is a product of environmental and proinflammatory stimuli and plays a key role in the initiation and persistence of airway inflammation (141). Tezepelumab by targeting TSLP, blocks the upstream signaling pathway of the inflammatory cascade, inhibiting the release of downstream type 2 inflammatory factors such as IL-4, IL-5, and IL-13, and has shown good results in severe asthma (142). A 52-week phase III trial demonstrated that subcutaneous tezepelumab 210 mg administered every 4 weeks as add-on therapy significantly reduced acute exacerbation rates and improved lung function, asthma control, and health-related quality of life compared with placebo in patients aged ≥12 years with severe asthma (143). Also, another phase III WAYPOINT trial further demonstrated that tezepelumab combined with standard therapy significantly reduced nasal polyp volume, improved olfactory loss scores (1-point reduction, *P* < 0.001), and decreased the proportion of patients requiring surgical intervention or systemic corticosteroids (0.5% vs. 22.1% in the placebo group) (144).

Benralizumab, an anti-IL-5 receptor alpha monoclonal antibody that induces eosinophil apoptosis, is FDA-approved for severe eosinophilic asthma and holds orphan drug status for eosinophilic EGPA (145, 146). While clinical evidence in CRSwNP remains limited, a recent Phase III trial demonstrated its efficacy, with benralizumab significantly improving NPS and NCS at week 40 compared with placebo (147).

Tralokinumab, a fully human anti-IL-13 monoclonal antibody approved for atopic dermatitis, may alleviate mucosal hyperplasia in CRSwNP by suppressing IL-13-driven inflammation, such as mucus hypersecretion and fibrosis (148). Similarly, lebrikizumab, another IL-13 inhibitor with notable efficacy in atopic dermatitis (over 50% achieving EASI-75 at 16 weeks as monotherapy), could target IL-13-mediated inflammation in CRSwNP (149).

Although these agents target key nodes in type 2 inflammation (e.g., TSLP, IL-5, IL-13) and expand possibilities for endotype-driven therapy, their efficacy and safety in CRSwNP require further clinical validation.

In recent years, clinicians have increasingly focused on the application of biologics in the treatment of CRSwNP (150). However, to date, no consensus has been reached regarding the optimal timing of administration or the selection of biologics for CRSwNP. Further research is needed to analyze additional clinical and molecular biomarkers, treatment efficacy, and adverse effects at baseline and follow-up time points, with the goal of predicting patient responses to biologic therapies in CRSwNP.

## 8 Conclusion and outlook

This review comprehensively discusses the pathophysiological mechanisms and emerging therapies for type 2 CRSwNP. The synergistic interaction between Th2 cells and ILC2s, mediated by IL-4/IL-13, establishes a positive feedback loop that drives eosinophilic infiltration and tissue remodeling. Dysregulation of the PD-1/PD-L1 immune checkpoint further exacerbates Th2 inflammation. Epithelial barrier dysfunction, characterized by a downregulation of tight junction proteins and impaired mucociliary clearance, facilitates pathogen penetration and bacterial colonization, while IL-13-induced overexpression of MUC5AC aggravates mucus retention. EMT, marked by reduced E-cadherin and elevated vimentin, promotes stromal transformation and is closely associated with basement membrane thickening and pseudocyst formation, particularly in eosinophilic CRSwNP. M2 macrophages recruit eosinophils via CCL-24 and synergize with Staphylococcus aureus superantigens to amplify Th2 inflammation and biofilm formation, contributing to disease recalcitrance. Among biologics, dupilumab demonstrates superior efficacy in reducing nasal polyp scores and restoring olfactory function, while mepolizumab and omalizumab offer personalized options for distinct endotypes (151).

There are certain limitations of this review. First, the heterogeneity of CRSwNP endotypes and the lack of standardized classification across studies may introduce bias in summarizing pathogenic mechanisms and treatment outcomes. For example, while eosinophilic inflammation is emphasized, non-eosinophilic subtypes, which are more prevalent in Asian populations, may involve distinct pathways not fully addressed here (152). Furthermore, the majority of clinical trials on biologics were conducted in Western populations, potentially limiting the generalizability of findings to other ethnic groups, particularly given the rising eosinophilic trends in Asian CRSwNP patients. In addition, the review focuses on type 2 inflammation but may underemphasize emerging non-type 2 mechanisms, such as neutrophilic inflammation and Th17 pathways, which warrant further exploration in future studies (153). Last, the absence of long-term safety data for biologics (e.g., >5 years) and real-world evidence from diverse healthcare settings highlights the need for extended follow-up and global multicenter trials.

Several conflicting findings also exist in the literature. For instance, while ILC2s are widely recognized as critical drivers of type 2 inflammation, some studies suggest that their role may vary depending on disease chronicity and tissue microenvironment. Stevens and Kato reported a 100-fold increase in ILC2s in nasal polyps, whereas others have observed context-dependent activation, with epithelial-derived IL-33 being essential in early inflammation but less so in chronic stages (28). This discrepancy underscores the need for longitudinal studies to clarify ILC2 dynamics. Another debate revolves around the biologics that despite the therapeutic potential of biologics targeting specific immune pathways, their clinical application faces fundamental challenges rooted in poly-pathogenic mechanisms and heterogeneous drug resistance (154). In CRSwNP, mepolizumab effectively suppresses IL-5-mediated eosinophil activation, yet persistent IL-33/TSLP signaling in local microenvironments may drive residual inflammation, leading to partial therapeutic responses. Furthermore, pharmacokinetic heterogeneity and immunogenicity risks contribute to secondary treatment failure. Therefore, even though biologics demonstrate significant short-term efficacy, their relatively brief history of clinical application, combined with insufficient long-term continuous observation and follow-up studies, have impeded their widespread adoption. Some studies may lead to underestimation or overestimation of efficacy and underreporting of important adverse events due to limited data volume and follow-up time. Moreover, the definition and evaluation of AEs are standardized and vary from study to study (155). Furthermore, establishing dosing regimens, administration schedules, and optimal treatment intervals is crucial, while these protocols must simultaneously balance therapeutic effectiveness, patient safety, and economic considerations. Long-term use of biological agents such as IL-4Rα inhibitors (e.g., dupilumab) or IL-5/IL-13-targeted formulations that may inhibit immune surveillance and increase susceptibility to infection (e.g., tuberculosis reactivation, bacterial/fungal infection), also theoretically raise the risk of malignancy, although the current evidence is inconclusive (156). The role of M2 macrophages also exhibits conflicting evidence. While some studies highlight their proinflammatory effects via CCL-24 secretion, others suggest that they may promote tissue repair through IL-10 production (157). This duality complicates the development of macrophage-targeted therapies.

It is widely recognized that most currently available biologics primarily target type 2 inflammatory pathways, but emerging evidence suggests that type 1 and type 3 inflammation also contribute to refractory CRSwNP (158). Future research must continue to elucidate additional clinically actionable unique biomarkers in CRSwNP that may serve as potential therapeutic intervention targets (159). Furthermore, the synergistic potential between biologics and endoscopic sinus surgery remains an area of active investigation requiring further exploration. Current guidelines recommend surgical intervention as first-line treatment in most cases prior to initiating biologic therapy, with biologic use in non-surgical candidates remaining relatively limited (160). Nevertheless, the anticipated reduction in biologic costs and growing patient preference for non-surgical management may lead to increased utilization of biologics in non-operative populations (161). Consequently, dedicated research into optimized coordination strategies between surgical and biologic interventions is warranted to establish integrated treatment protocols.

Although biologics have revolutionized CRSwNP treatment, particularly for type 2 CRSwNP, their long-term safety, cost-effectiveness, and individualized application require further validation through large-scale real-life studies. Therefore, future research directions should focus on integrating multiomics data to decipher disease heterogeneity, identifying precise pathogenic mechanisms, and developing AI-driven predictive models for therapeutic response stratification across different endotypes, while simultaneously incorporating environmental, social, and genetic factors into the analysis of CRSwNP pathogenesis. This will ultimately enable a paradigm shift from phenotype-driven to endotype-driven precision medicine, optimizing outcomes for patients with CRSwNP.

## Glossary

AEs: adverse events
AR: allergic rhinitis
CLPs: common lymphoid progenitors
CRS: chronic rhinosinusitis
CRSsNP: chronic rhinosinusitis without nasal polyps
CRSwNP: chronic rhinosinusitis with nasal polyps
DC: dendritic cell
ECP: eosinophil cationic protein
ECRSwNP: eosinophilic CRSwNP
EGPA: eosinophilic granulomatosis with polyangiitis
EMA: European Medicines Agency
EMT: epithelial–mesenchymal transition
FDA: Food and Drug Administration
FeNO: fractional exhaled nitric oxide
HES: hypereosinophilic syndrome
ILC2s: type 2 innate lymphoid cells
LCR: locus control region
LMS: Lund–Mackay score
MCC: mucociliary clearance
MIF: migration inhibitory factor
MMPs: matrix metalloproteinases
MPO: myeloperoxidase
NCS: nasal congestion score
NPS: nasal polyp score
NPs: nasal polyps
PD-1: programmed cell death protein 1
PD-L1: programmed death-ligand 1
RCTs: randomized controlled trials
RSOM-31: Rhinosinusitis Outcomes Measure-31
SCC: systemic corticosteroids
SEA: severe eosinophilic asthma
SNOT-22: Sino-Nasal Outcome Test-22
Spl: Serine protease-like protein
TER: transepithelial electrical resistance
TGF-β1: transforming growth factor-β1
Th: T helper cells
Th2: Type 2T helper cells
TJs: tight junctions
Treg: regulatory T cells
TSLP: thymic stromal lymphopoietin
VEGF: vascular endothelial growth factor
