# Correction: The advance on pathophysiological mechanisms of type 2 chronic rhinosinusitis with nasal polyposis

**DOI:** 10.3389/falgy.2025.1679519

**Published:** 2025-09-02

**Authors:** Cheng Yang, Ling Guo, Yuhan Wang, Wenjing Jiang, Sijia Chen, Qingjia Gu

**Affiliations:** 1 Department of Otolaryngology Head and Neck Surgery, Sichuan Provincial People’s Hospital, University of Electronic Science and Technology of China, Chengdu, China; 2 Department of Otolaryngology Head and Neck Surgery, Sichuan Provincial People’s Hospital, Chengdu University of Traditional Chinese Medicine, Chengdu, China

**Keywords:** chronic rhinosinusitis with nasal polyposis (CRSwNP), type 2 T helper cells (Th2), type 2 innate lymphoid cells (ILC2s), epithelial barrier dysfunction, biologics

The figures were in the wrong order in the published version of this article. Figures 1 and 2 were inadvertently swapped, while their respective legends remain correctly positioned. The order has now been corrected.

The original version of this article has been updated.

